# Commentary: The safety and efficacy of balloon-expandable vs. self-expanding trans-catheter aortic valve replacement in high-risk patients with severe symptomatic aortic stenosis

**DOI:** 10.3389/fcvm.2023.1295274

**Published:** 2023-12-18

**Authors:** Michael J. Reardon, Tanvir Bajwa, Jeffrey J. Popma

**Affiliations:** ^1^ Houston Methodist-DeBakey Heart and Vascular Center, Houston, TX, United States; ^2^ Aurora St. Luke’s Medical Center, Milwaukee, WI, United States; ^3^ Medtronic, Minneapolis, MN, United States

**Keywords:** TAVR, TAVI, balloon-expandable, self-expanding, aortic stenosis, randomized controlled trial

A Commentary on The safety and efficacy of balloon-expandable vs. self-expanding trans-catheter aortic valve replacement in high-risk patients with severe symptomatic aortic stenosis By Senguttuvan NB, Bhatt H, Balakrishnan VK, Krishnamoorthy P, Goel S, Reddy PMK, Subramanian V, Claessen BE, Kumar A, Majmundar M, Ro R, Lerakis S, Jayaraj R, Kalra A, Flather M, Dangas G, Tang GHL (2023). Front. Cardiovasc. Med. 10: doi: 10.3389/fcvm.2023.1130354

**Figure 1:**
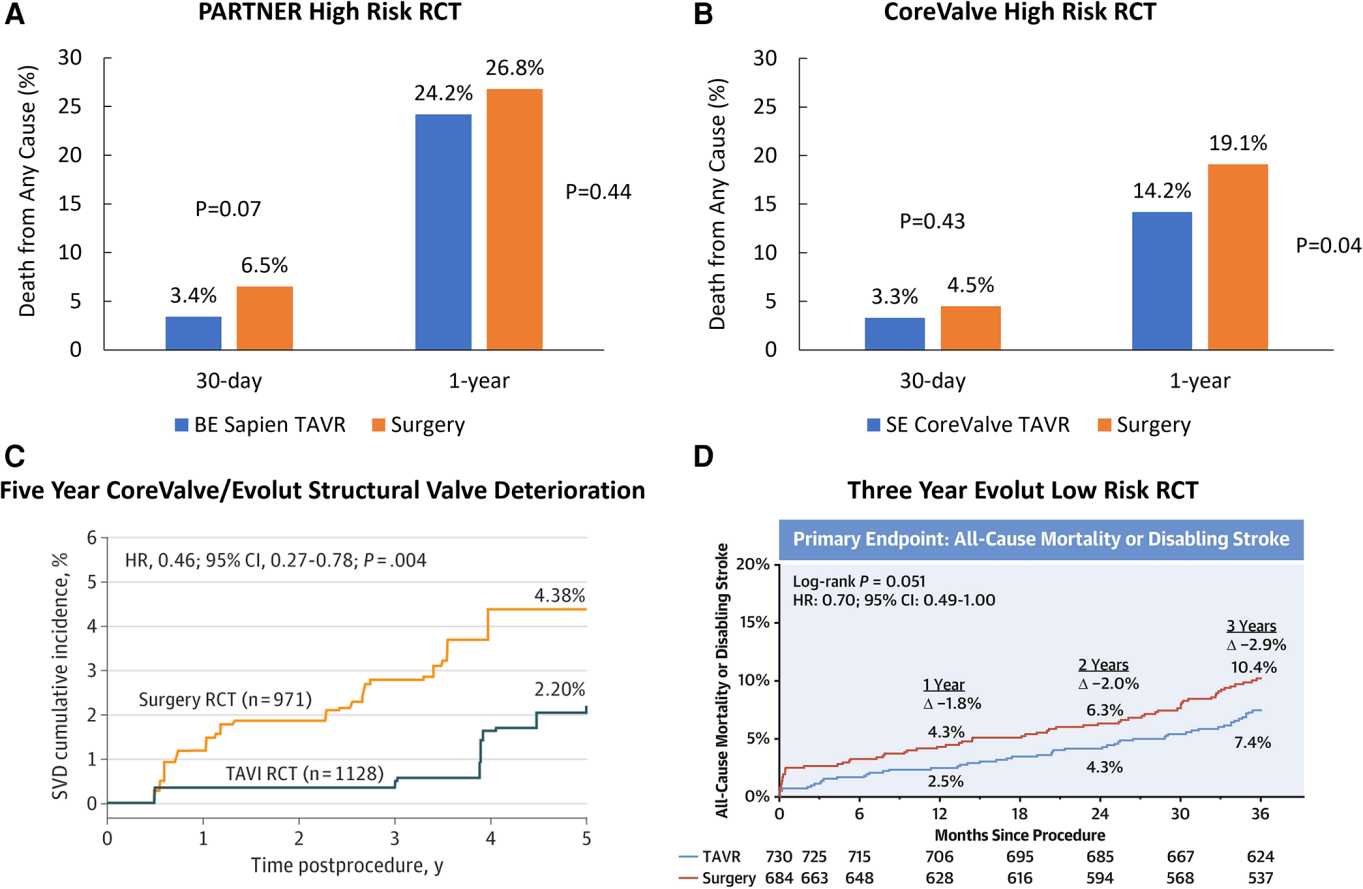
Randomized controlled trials comparing TAVR with surgery. (**A**) Primary endpoint of the PARTNER 1 A randomized controlled trial in high surgical risk patients. (**B**) Primary endpoint of the CoreValve high risk randomized controlled trial in high surgical risk patients. (**C**) Rates of structural valve deterioration in patients treated with CoreValve/Evolut TAVR or surgery. Reproduced with permission from O'Hair D, Yakubov SJ, Grubb KJ, Oh JK, Ito S, Deeb GM, et al. Structural Valve Deterioration After Self-Expanding Transcatheter or Surgical Aortic Valve Implantation in Patients at Intermediate or High Risk. JAMA Cardiol. 2022. Copyright© 2022 American Medical Association. All rights reserved. (**D**) Three Year primary endpoint in the Evolut low risk randomized controlled trial. Reprinted from Forrest JK, Deeb GM, Yakubov SJ, Gada H, Mumtaz MA, Ramlawi B, et al. 3-Year Outcomes After Transcatheter or Surgical Aortic Valve Replacement in Low-Risk Patients With Aortic Stenosis. J Am Coll Cardiol. 2023;81(17):1663–74, with permission from Elsevier.

We read with interest the meta-analysis of Senguttuvan and colleagues (1) that concludes that balloon-expandable (BE) transcatheter aortic valve replacement (TAVR) is associated with reduced all-cause mortality and cardiovascular mortality at 30 days compared to self-expanding (SE) TAVR in high surgical risk patients. Their conclusions underscore the inherent limitations of meta-analyses, such as mixing historical randomized controlled trials (RCTs) and registries that are nearly a decade old, use of early term (30-day) rather than long term (up to 5 years) outcomes, and incomplete propensity matching of very different risk populations. Most importantly, the authors grouped all SE bioprostheses together, when, in fact, there may be important differences amongst SE devices. For example, a network analysis of BE Sapien TAVR (Edwards Lifesciences, USA) and SE CoreValve/Evolut TAVR (Medtronic, USA) RCTs in high risk patients would use surgery as the comparator group for both devices. The BE Sapien PARTNER 1A RCT in high surgical risk patients showed similar rates of 30-day (TAVR, 3.4%; surgery, 6.5%; *P* = 0.07) and 1-year (TAVR, 24.2%; surgery, 26.8%; *P* = 0.44) all-cause mortality in the two groups (Figure 1A) (2). The SE CoreValve high surgical risk RCT found similar rates of 30-day mortality (TAVR, 3.3%; surgery, 4.5%; *P* = 0.43), but a statistically 1-year *lower* mortality with TAVR compared with surgery (TAVR, 14.2%; surgery, 19.1%; *P* = 0.04) (Figure 1B) (3).

With respect to long-term valve durability, the 5-year outcomes of the PARTNER IIA RCT in patients at intermediate surgical risk found similar rates of structural valve deterioration (SVD) between BE Sapien 3 and surgery (TAVR, 3.9%; surgery 3.5%; *P* = 0.65), and a higher rate of SVD with BE Sapien XT (TAVR, 9.5%; surgery 3.5%; *P* < 0.001) (4). The 5-year durability post-hoc analysis performed in similar intermediate and high risk patients showed *lower* rates of SVD with SE CoreValve/Evolut TAVR compared to surgery (TAVR, 2.2%; surgery, 4.4%; *P* = 0.004) (5) (Figure 1C). In lower surgical risk patients, there were no differences in death or disabling stroke in patients treated with BE Sapien 3 vs. surgery at 2 years (TAVR 3.0%; surgery 3.8%; *P* = 0.47), although valve thrombosis at 2 years was higher after TAVR (TAVR 2.6%; surgery, 0.7%; *P* = 0.02) (6). The Evolut low risk RCT found a numerically lower rate of all-cause mortality or disabling stroke in patients treated with SE Evolut TAVR compared to surgery at 3 years (TAVR, 7.4%; surgery, 10.4%; *P* = 0.051). (Figure 1D) (7).

Nevertheless, these comparative RCT findings vs. surgery as the common denominator are not conclusive, underscoring the need for specific device-device RCTs. The SMART trial (ClinicalTrials.gov Identifier: NCT04464421) has completed randomization of 700 patients with a small (<430 mm^2^) aortic annulus treated with BE Sapien 3 or SE Evolut TAVR, and will compare valve performance at 1 and 5 years. More direct comparative analyses are needed before drawing any conclusions that one class of device is safer than another from these types of meta-analyses, particularly those that are not concordant with prospective RCTs. Meta-analysis as a statistical combination of the outcomes of different trials is limited by the quality of the studies included, the heterogeneity of the individual studies, as well as potential publication bias.
